# The sepsis journey and where digital alerts can help: a qualitative, interview study with survivors and family members in England

**DOI:** 10.3389/fpubh.2025.1521761

**Published:** 2025-03-26

**Authors:** Runa Lazzarino, Aleksandra J. Borek, Andrew J. Brent, John Welch, Kate Honeyford, Ron Daniels, Anne Kinderlerer, Graham Cooke, Shashank Patil, Anthony Gordon, Philippa Goodman, Ben Glampson, Peter Ghazal, Ceire Costelloe, Sarah Tonkin-Crine

**Affiliations:** ^1^Nuffield Department of Primary Care Health Sciences, Medical Sciences Division, University of Oxford, Oxford, United Kingdom; ^2^National Institute for Health and Care Research (NIHR) Health Protection Research Unit in Healthcare Associated Infections and Antimicrobial Resistance, University of Oxford, Oxford, United Kingdom; ^3^Oxford University Hospitals NHS Foundation Trust, Oxford, United Kingdom; ^4^University College London Hospitals NHS Foundation Trust, London, United Kingdom; ^5^NIHR Central London Patient Safety Research Collaboration, London, United Kingdom; ^6^Team Health Informatics, Institute of Cancer Research, London, United Kingdom; ^7^Global Business School for Health, University College London, London, United Kingdom; ^8^UK Sepsis Trust and Global Sepsis Alliance, Birmingham, United Kingdom; ^9^University Hospitals Birmingham NHS Foundation Trust, Birmingham, United Kingdom; ^10^Imperial College Healthcare NHS Trust, London, United Kingdom; ^11^Chelsea and Westminster Hospital, London, United Kingdom; ^12^PPI Representative, Oxford, United Kingdom; ^13^School of Medicine, Cardiff University, Cardiff, United Kingdom; ^14^School of Public Health, Imperial College London, London, United Kingdom

**Keywords:** sepsis survivors, family members, digital alerts, secondary emergency care, England

## Abstract

**Introduction:**

The fight against sepsis is an ongoing healthcare challenge, where digital tools are increasingly used with some promising results. The experience of survivors and their family members can help optimize digital alerts for sepsis/deterioration. This study pairs the experiences of survivors of their sepsis journey and family members with their knowledge and views on the role of digital alerts.

**Methods:**

A qualitative study with online, semi-structured interviews and focus groups with sepsis survivors and family members in England. Data were analyzed inductively using thematic analysis.

**Results:**

We included 11 survivors, and 5 family members recruited via sepsis charities and other social media, for a total of 15 sepsis cases. Identified categories correspond to the three stages of the sepsis journey: 1. Pre-hospital, onset symptoms and help-seeking; 2. Hospital admission and stay; 3. Post-sepsis syndrome. The role of digital alerts at each stage of the sepsis journey is discussed. Participants’ experiences were varied, previous sepsis awareness scant, and knowledge of digital alerts minimal. However, participants were confident in the potential of alerts contributing along the sepsis journey. They perceived digital alerts as important in healthcare professionals’ decision-making to expedite identification and treatment of sepsis and suggested their expansion across healthcare services. Participants expressed that awareness should be increased among the general public about digital alerts for sepsis/deterioration.

**Discussion:**

In light of sepsis’ insidious and variable manifestation, the involvement of patients and family members in the development of digital alerts is crucial to optimize their design and deployment towards improving outcomes. Digital alerts should enhance the connection across healthcare services as well as the care quality. They should also enhance the communication between patients and healthcare professionals.

**Clinical trial registration:**

The ClinicalTrials.gov registration identifier for this study is NCT05741801; the protocol ID is 16347.

## Introduction

Reducing the morbidity and mortality from sepsis is among the top, ongoing healthcare challenges on a global scale (1). Despite a declining trend, in 2017 nearly 49 million cases of sepsis were estimated worldwide (2). Sepsis is not equally distributed across world regions, with lower-resourced settings carrying the highest burden (2). Nonetheless, high-income countries are not immune. In the UK 48,500 sepsis-related deaths were estimated to occur yearly (3).

Sepsis is not a discrete disease. It is a dynamic, potentially overwhelming, dysregulated response to an infection, whereby the body starts attacking its own tissues, leading to organ dysfunction (4). Signs and symptoms are diverse and insidious, which risk sepsis going unnoticed or being misinterpreted. Sepsis demands prompt identification and treatment to facilitate better outcomes (5) and higher post-sepsis quality of life (6).

To date, qualitative research with sepsis survivors is scant. Studies have found that the ability to recognize the onset of sepsis is inadequate in the general public (7, 8). This hinders timely recognition of symptoms and care seeking (7). In a recent study on patients’ and family members’ stories of the onset of sepsis, people indicated that symptoms and signs manifested insidiously and then markedly worsened (9). Two other studies found that symptoms are mostly minor, varied, and often nonspecific before becoming critical – which may occur extremely rapidly (10, 11). Due to the deceptive and dynamic nature of sepsis’ signs and symptoms, often family members or carers were the ones suspecting the severity of the condition and initiating help seeking (9, 11–13).

In relation to ambulance and hospital settings and the experience of patients or professionals, literature is even scarcer. However, it similarly indicates that the polymorphous presentation of septic patients, the frequent presence of co-morbidities, and the reliance on self-reported symptoms hamper quick identification and management (14). With respect to prehospitalization, a study with ambulance paramedics found that previous experience of dealing with potentially septic patients influenced knowledge and assessment, contributing both to greater confidence but also to greater uncertainty – particularly when patients’ symptomatology was ‘out of the box’ (15).

Primary care recognition of sepsis has not received much attention either. A recent survey in general practices found that not all staff were adequately trained about sepsis, that several clinics had practical issues in relation to taking blood cultures, and that standardized guidance was needed in relation to intravenous antibiotic administration (16). General practitioners’ (GPs) assessment of potential serious infections was shown to be a complex process, where patients’ general appearance and history as well as a GP’s ‘gut feeling’ guided the decision of a hospital referral – in addition to the vital signs of the patient (17).

Literature exploring views and experiences of healthcare professionals (HCPs) in secondary care has highlighted limitations in professionals’ capacity and capability to identify sepsis. The main identified factors linked to these challenges were related to handover and escalation of patients in busy environments, as well as errors in communication across hospital teams (14, 15, 18–20).

A larger corpus of qualitative evidence involving patients and family members is on sepsis survivorhood. Post-sepsis syndrome, which has been increasingly investigated and recognized over the past two decades, is marked by increased risk of death and a poor health-related quality of life (6, 21). Similarly to post-intensive care syndrome (22), post-sepsis syndrome has been associated with worsening of physical functions (from daily chores to reading), psychological and neurocognitive conditions (with anxiety, depression and fatigue reported), and higher risks of medical conditions (such as cardiovascular disease and infection) and re-hospitalizations (23–25).

To better face the challenges that sepsis presents across all its phases, digital alerts for sepsis/deterioration have been incorporated into some hospitals’ electronic health records. There are different types of digital alerts, from passive icons on a computer screen to pop-up boxes interrupting users’ workflow until actioned (26). Alerts are based on different algorithms reflecting the different screening tools used for sepsis/deterioration (27, 28). Alerts based on machine learning are also increasingly trialed (29).

The growing evidence of the impact of digital alerts is promising, with studies demonstrating a positive association with timely treatment and reduction in mortality (30–32). Nonetheless, the available evidence still has important limitations. It mainly concentrates on hospital settings and quantitative measures of patient outcomes, and it is accompanied by little qualitative work on HCPs’ views and use of the alerts (33, 34). There is no qualitative work on patients’ views of digital alerts to support sepsis diagnosis and management.

This study aimed to explore (a) survivors’ and family members’ experiences of the sepsis episode, and (b) their views about the potential role of digital alerts for sepsis/deterioration.

## Methods

### Study design

This qualitative study was part of the Digital Alerting for Sepsis (DiAlS) study, a program of research aiming to evaluate the impact of digital alerts on patient outcomes and staff practice in NHS Hospital Trusts^1^. This paper presents and discusses results of the qualitative work with sepsis survivors and family members of sepsis survivors.

### Participants and sampling

We purposively sampled participants to identify and select adults who have had at least one sepsis episode treated in a hospital in England, or who were family members of someone who had had sepsis and was treated in hospital. The study recruitment advert was circulated on social media: Facebook, Twitter, and LinkedIn accounts of two of the researchers in the study (ST-C and RL), as well as the websites of the UK Sepsis Trust charity, the NIHR Be Part of Research portal, and the Nuffield Department of Primary Care Health Sciences of the University of Oxford. Two clinical co-investigators in the DiAlS study directly shared or asked other hospital staff to share the study advert with eligible ex-patients, either in person, by telephone, or by email. Eligible participants could choose between a focus group or a one-to-one interview, either online or over the phone, and were asked to contact the research team if they were interested in participating.

### Data collection

Data were collected between early December 2022 and mid-February 2023 by RL, using video-conferencing software (*Microsoft Teams*), besides one telephone interview. Focus groups and individual interviews were conducted using a semi-structured topic guide to ensure that key questions were asked to all participants but to allow flexibility for follow up questions. Participants were encouraged to talk about any topics which were of importance to them in relation to the research aims. The topic guide (Supplementary material) was developed from the study objectives with input from the wider research team and the study Patient and Public Involvement representative. Patients and family members were firstly asked about their previous experience of sepsis in hospital settings. The second part of the interview concentrated on participants’ views on digital alerts for sepsis/deterioration. The purpose, functioning, and use of the digital alerts were illustrated to interviewees, explaining also how and where they would normally appear in the hospital digital system and how HCPs could act upon an alert. Individual interviews and the focus group lasted approximately 1 house, were audio-recorded, and professionally anonymized and transcribed. Participants were offered a £20 voucher in recognition of their time and contribution, and this was shared with an accompanying email where relevant support charities and groups for sepsis survivors were listed. Data collection closed when inductive thematic saturation was considered reached as no new themes were identified by data analysis (35).

### Data analysis

Data analysis was led by RL and started while data collection was ongoing, with the support of *NVivo 12*. An inductive approach to thematic analysis was undertaken (36, 37). Codes were compared with one another to create unique categories, grouping similar codes together. Subsequently, categories were grouped into higher-level categories. We used investigator triangulation with two senior researchers (AJBorek and ST-C) analyzing and coding two different interview transcripts each to inform the development of the coding framework. We held frequent discussions during the coding and analytic process to identify and organize the findings in the most meaningful way. We kept records of the data analysis process. The quality criteria for qualitative research informed our process to ensure rigor and trustworthiness (38).

### Ethics

The DiAlS Study was approved by the UK NHS Health Research Authority (Project ID - 288328). Separately, the qualitative work stream was reviewed and approved by the Research Governance, Ethics & Assurance Team (RGEA) of the University of Oxford and the UK NHS Health Research Authority of England and Wales (Project ID 313699–22/PR/1020). Full, recorded verbal consent was obtained from all participants.

## Results

We conducted 11 individual interviews, 1 paired interview, and 1 focus group with three family members – covering 15 sepsis cases. In total, we included 16 participants, of whom 11 were sepsis survivors and 5 family members (Table 1). All participants had completed secondary education, or above, and 7/16 had an occupation in the healthcare sector. Nearly half of the survivors had sepsis in the previous 4 years, and 8 cases were urosepsis or involving the pelvic areas. Two survivors had to retire early or reduce their commitment to work due to sepsis sequelae.

**Table 1:** Main characteristics of participants.

	Participants (*n*)	Declared gender (*n* = female)	Age in years; mean (range)	Ethnic background (*n* = White British)	Education (*n* = secondary education only)	Treated in a teaching hospital (*n*)	Aware of digital alerts in general (*n*)	Aware of sepsis digital alerts (*n*)
Sepsis Survivors	11	10	45.6 (21–74)	11	4	4	2	1
Family Members	5	4	48.6 (43–56)	5	1	2	2	0
Total	*N* = 16	*N* = 14	*M* = 47	*N* = 16	*N* = 5	*N* = 6	*N* = 4	*N* = 1

Based on an online search to check whether the hospitals where the participants were treated had a digital system in place at time of admission, six hospitals appeared to ‘have gone digital’ with electronic patient records, but only two had a digital alert for sepsis/deterioration specifically. Only one participant, the youngest, treated in one of these two hospitals, declared being aware of a digital alert for sepsis being in use.

Below we present the main findings according to the three main, discrete stages of the sepsis journey: (1) Pre-hospital, onset symptoms and help-seeking; (2) Hospital admission and stay; (3) Post-sepsis syndrome. At the end of each section, we present results related to digital alerts for sepsis/deterioration which were relevant to that specific stage of the sepsis journey.

### Stage 1: pre-hospital, onset symptoms and help-seeking

Participants described their sepsis onset symptoms both in subjective (e.g., something unprecedented, excruciating pain, impending doom, as drunk) and objective (e.g., not passing urine, high temperature, skin rash) terms. For a few survivors, the deterioration process was long, spanning over a week, whereas for others, it lasted only a few hours before reaching a peak and entailing emergency and intensive care at the hospital. In spite of these variations, all participants said that they found it difficult to realize that they were on a deterioration pathway leading to a state of acuity; this is because they found their initial symptoms hard to interpret, camouflaging as tiredness or a common cold.

*When it first happened, when I first started feeling unwell, I thought I’ve just got the flu or something. Actually, first of all I thought I was dehydrated. […] Then I was shaking. This is not right. Something’s wrong with me. As I said, I had this feeling of impending doom. This is my brain tricking me. I was just so confused about everything.* (Survivor 1, 21 years)

Significantly, difficulties in suspecting any severe deterioration were reported also by participants who were HCPs and had some knowledge of infection and sepsis. Some of those who knew about sepsis emphasized that they held a distorted understanding of it, as an extraordinary and inevitably fatal condition, associated with septicemia or sepsis shock, and only affecting older adults. After their episode, all participants underlined that sepsis can present much more subtly and ‘can hit anyone’. In many situations described by participants, the support of those around them was crucial, in terms of accelerating or taking the lead on professional help seeking.

*I just generally felt really washed out and tired. Nothing really to put a finger on it. […] I don’t know how much longer after and my behavior had changed, in the sense that I just felt sick, so he helped me to the bathroom. I was sick. I needed to have a shower, so he helped me in the shower. Then he walked me back to the bed. He said I was acting really odd. I went to get on the bed and that’s the moment that I collapsed on the bed and just became… I was unresponsive, so he called the ambulance.* (Survivor 9, 55 years)

Depending on the severity of their initial symptoms, participants recount calling either 111 or 999 or their GP. Participants’ experiences of these calls varied, and some perceived the conversations and the following health tests and course of actions undertaken as lifesaving and thorough. By contrast, other participants felt not adequately listened to by HCPs and some attributed this to a lack of awareness and preparedness in relation to sepsis.

*My daughter said, “Oh I thought that was the school toilets that smelt like that.” She hadn’t realized it was her [urine] […]. The next morning, I got an appointment at the GP’s. […] I think this is why I’m so keen on the alert for sepsis. Because I said I thought she’d got a wee infection, that was what the GP logged onto. He was like, have some antibiotics, go home.* (Family member 8, daughter had sepsis at 11 years old)

Those participants who were self-monitoring and self-tracking their health data found this useful in terms of becoming more quickly aware that their condition was out of the norm. Some participants were wearing fitness trackers, whereas others self-tested with blood pressure monitors and thermometers; they found it helpful to have health data at hand which triggered help-seeking and gave them some objective parameters to report to the ambulance and primary care staff. Several participants also voiced the wish that more digital resources should be developed and made available to the general public to use at home to facilitate the self-identification of acute illness, including sepsis.

*Then on the Friday morning, and I’d literally just got out of bed. Walked to my en-suite bathroom, which is five paces and my fitness tracker, again, was alerting me to this high heart rate. It had been doing it all week and I just kept taking deep breaths and trying to slow it down and so I thought maybe I should phone my GP. […] It was my fitness tracker that saved my life.* (Survivor 3, 60 years)

Some participants felt that digital alerts for sepsis had potential for community settings and that general practice, 111 call handlers and paramedics may all be able to use such an alert system in their roles to more quickly identify sepsis.

*It’s that initial primary care… that’s where my concern is, that if someone does go to a GP and they aren’t advised correctly, then they miss out potentially in getting to secondary care where these alerts do work.* (Family Member 6, relative had sepsis at 1 month)

*If you phone 111 do they have the digital alert system, so a call handler on the end of [the line], if I was to have rung up and described the symptoms that my daughter had, would that have triggered to them that it was more than a cold? The same with the ambulance crew.* (Family member 8, relative had sepsis at 11 years)

### Stage 2: hospital admission and stay

Participants conveyed varied experiences with regards to the healthcare they received in hospitals. For some survivors, ED assessment, triaging, and admission constituted a quick process. Depending again on the severity of their symptoms at presentation, some participants were admitted to an ED resuscitation unit or straight to intensive care. Others, among those who self-presented via the walk-in route, lamented that they had to wait long hours in the ED reception area. This latter sub-group attributed the sub-optimal care received to the unpreparedness of the triaging nurses and to the busyness of the ED environment. The majority of those who reported a negative experience at admission had their sepsis episode during the peak of the COVID-19 pandemic, the weekend, in the evening, at night, or during a festivity – such as Halloween parties in the experience of Survivor 10:

*There were people on trolleys everywhere – there were barely enough seats for people to sit […]. The bloods that had been taken – that they hadn’t actually waited to come back before discharging me – my CRP was 350, so I should absolutely not have been discharged! But I appreciate that those things happen when A&E is under that much pressure.* (Survivor 10, 24 years)

Hospitalization in moments of increased busyness and decreased presence of senior decision makers seems to have contributed to a negative experience during the hospital stay too. Some bemoaned sub-optimal patient management, raising issues such as very long waiting times for test results or for an operating theatre, frequent handovers, the administration of wrong treatment, or multiple changes or missed doses of treatment, and consecutive surgeries. However, most participants felt grateful for how their condition was managed in the hospital, particularly in the intensive care unit where most described receiving excellent care. Family members and those survivors who could retain some memories of those critical moments described the clinical observations, the tests, and the treatment administered, pointing to how the HCPs were working in a team, across hospital units, and even across different hospitals, first towards an accurate diagnosis, and second for optimal management.

At the same time, other participants perceived the clinical teamwork towards identifying the source of the infection and initiating the right treatment as a tortuous process. To some participants, HCPs seemed to be operating more using a ‘trial-and-error’ approach. Other participants felt that they were affected by the perceived uncertainty of their clinical team, and even more when they could note discord within the team. Uncertainty and discord triggered a sense of distrust and anxiety in them.

*They kept trying to grow the cultures to find out what the bug was, but they kept me on a wide spectrum of antibiotics. From what I can guess it was a bit of a… it seemed to me like it was a bit of a guess, a bit more of an art than a science […] it wasn’t like they’d run a blood test and it should have been this or that, it was like, right try reducing this drug a little bit* (Family member 16, 56 years)

Finally, some participants felt that the care they received during their hospital stay was lacking humaneness. They voiced this view with reference to the communication with the clinical team, which they experienced as hasty, not exhaustive or informative enough, and not reassuring or compassionate. These participants felt disempowered and not listened to as some just overheard, had to guess, or only read ‘sepsis’ in their discharge papers for the first time.

*My lead consultant came […] down and said, “there’s nothing wrong with her. There’s literally nothing wrong with her. She’s fine.” I only found out afterwards, because the junior doctor that was there that day said to me, that my consultant was telling them outside just to leave me and there’s nothing wrong with me. I’m just being a drama queen.* (Survivor 1, 21 years)

On the contrary, for other participants, the communication with HCPs was more straightforward and reassuring.

A few participants knew about the existence of digital alerts for sepsis in hospitals and expressed that more awareness and information on how they work and how they are used would be reassuring for patients. Additionally, some participants wished that patients and family members were more involved in consultations on how to develop such digital alerts. Both survivors and family members were of the opinion that no algorithm or digital alert should substitute human clinical expertise and judgment based on visiting the patient in person. They also thought that efficient teamwork and training were indispensable to improve sepsis identification and prompt management. Many participants viewed digital alerts positively as useful tools helping HCPs speed up clinical diagnosis and appropriate treatment, and consequently improve patient outcomes. For this, some participants specified that digital alerts should flag to all the relevant teams in the hospital – from the triage nurse to the microbiology team – which they felt would facilitate teamwork and reduce the risk of missing a patient.

*The [digital alert] should reach everyone that you can and make them aware that you are at risk, again through education, making it more knowledgeable and user-friendly as well, just to keep everybody in the same loop, really, like we do at work. We have handovers, there’s constant continuity in a practice where we’re thinking and working together, and I think, to do that digitally, well, what can go wrong, really?* (Survivor 4, 31 years)

A few participants envisioned digital alerts being very specific and tailored, both in relation to the patient and the HCP they flag to. Accordingly, they felt they should be portable/wearable and be devised to convey detailed information of the patient. The only participant who was knowledgeable about sepsis digital alerts commented:

*I feel it [digital alert] needs to be more tailored because right now it’s almost too generic. It’s too generic to be as good as it can be. […] I think patients should know that they exist. It is sort of a reassurance for people.* (Survivor 1, 21 years)

Other participants echoed the importance of raising the awareness among the general public and patients about digital alerts for sepsis/deterioration. Finally, a number of participants emphasized that digital alerts should factor in previous sepsis episodes, less common symptoms in addition to key parameters, and what patients are expressing and how they are feeling. In this sense, participants suggested that digital alerts should enhance and not reduce patient-HCP communication.

*I think, first symptoms that are included – obviously, things like blood pressure – blood tests – general observations – but it should also be what the patient’s telling you – you know, whether they’ve been able to urinate – whether they’re shivering and shaking – how they generally feel in themselves, ‘cause I knew – and I’ve had other people say – ‘I knew something was wrong’ – and I couldn’t say to you, ‘Oh, …’ – like with a broken arm – ‘My arm hurts – that’s what’s wrong,’ but I knew something was wrong, and it wasn’t good.* (Survivor 12, 42 years)

### Stage 3: post-sepsis syndrome

Participants’ reported sepsis sequelae ranged from mental health and cognitive issues (e.g., memory loss, ‘sepsis cloud’, health anxiety, depression) to physical impairments (e.g., vision reduced, fatigue, headaches).

*The other thing is the aftercare. We had no support. If I had been having some therapy during that time when he was really ill in that ITU, maybe I wouldn’t have been suffering from post-traumatic stress disorder now […]. Obviously, we thought we were managing fine. Two months later, I was actually driving in the direction of the hospital and I just had a massive flashback and a panic attack and I had to pull over, and that’s when I first realized I was suffering from post-traumatic stress disorder. I then spoke to my GP and got some counselling. […] My son then, three months after I was diagnosed with post-traumatic stress disorder, attempted suicide.* (Family member 14, son had sepsis at 12 years old)

The stage after the sepsis episode was where both survivors and family members felt least supported and that they lacked knowledge. Most participants complained that they received very little information, de-briefing, and follow-up support from the healthcare system. In fact, while only a few received post-intensive care help, the majority turned to, and found remarkable support in, charities and peer-support groups outside of the medical sphere.

*They didn’t talk to me about sepsis whatsoever. When I finally got discharged, I was just discharged and told I would get a dermatology appointment to look at my foot. I was given no information about sepsis whatsoever. I thought that once I was over it, I was over it. I didn't realize that I would still be feeling not great sort of months down the line.* (Survivor 2, 45 years)

Participants suggested ways to better meet the support needs in survivorhood, including dedicated sepsis teams running aftercare clinics that could incorporate digital alerts in their practice as well as other digital ways to follow-up and involve survivors along their recovery pathway. These sepsis teams – other participants suggested – should also promote and deliver staff education and training; raise awareness about sepsis and post-sepsis syndrome; and facilitate peer support, for example with sepsis cafés – as one participant mentioned.

*The hospital where I went first of all, where the doctor told me to go home, a couple of nurses run a sepsis café. It just popped up on my phone – obviously, it must have heard me talking about it – so probably about a year after I’d had it, it popped up and it’s like every other month and you go there for a set time, and you can go and talk to them, you can talk to other people who’ve had it, which is really helpful, because you don’t feel like you’re on your own, that other people have had it, and we’ve all had it from different ways.* (Survivor 13, 53 years)

Participants felt that the introduction and use of sepsis digital alerts should go hand-in-hand with a ‘massive’ increase in resources for the NHS for awareness raising and the involvement of the general public, in particular sepsis experts by experience.

*It is just about education, getting it into schools, getting it into First Aid. […] Teaching people from a younger age and First Aiders what the first signs of sepsis are, and it’s great to see them on ambulances, but […] until you’ve spoken to somebody, or had it, or a relative, you don’t realize how big it is in somebody’s life until it happens, so I think the biggest thing from this, along with the digital alerts, is just about knowledge.* (Survivor 4, 31 years)

In addition to general awareness raising, professional staff education, targeting those HCPs spending more time at the patient’s bedside, was mentioned by a few participants as a priority, and for this, they suggested that sepsis was introduced in the curriculum of all levels of nursing training.

## Discussion

Awareness of digital alerts for sepsis/deterioration was minimal in our sample, nonetheless, participants viewed alerts as useful in all the three stages of the sepsis journey. Participants expressed that digital alerts should be made more available, both among the general population, such as in wearable fitness trackers, and in primary health, telephone and ambulance services. Participants saw digital alerts in the hospital as potentially advantageous partners, but never substitutes, of HCPs’ clinical judgment. Some participants maintained that digital alerts could be useful in aftercare, for patients’ self-monitoring and aftercare clinics as tools to monitor and follow-up survivors along their recovery. Some participants also expressed how important it would be that digital alerts were developed with experts-by-experience and factored in less common symptoms and patients’ illness experience so to foster communication and patient-centered care.

Knowledge about sepsis was scarce and incomplete across the sample, associated with older adulthood and with the most acute stage of sepsis. The role of family members in initiating professional help-seeking was decisive and life-saving in many cases. Some participants recalled the practice of the ambulance service and their GPs as accurate and fast; whereas others reported dismissiveness and perceived shortcomings that they attributed to lack of preparedness to promptly identify and treat sepsis. Participants’ experiences of their hospital admission and stay were also diverse. The worst experiences were ascribed to busyness of the admission units. Opinions on lack of communication were voiced in relation to post-sepsis syndrome, together with a widely shared complaint about deficient sepsis aftercare.

Our results resonate with previous qualitative research with sepsis survivors, building on this to understand patient and family perspectives on sepsis/deterioration digital alerts. Studies have reported on patients’ decisions to seek help with symptoms, experiences of hospitalization, and how patients have managed life after surviving sepsis (7, 10, 11). Rooted in the experience and the views of sepsis survivors and family members, our work indicates that digital alerts have a further untapped potential in all phases of the sepsis journey.

Our participants suggested a potential role of digital alerts for sepsis out of the hospital, including at home, in the ambulance, and general practice. As in other studies, participants were uncertain and did not easily attribute their symptoms to sepsis (9, 13). For this, they expressed the view that digital alerts for sepsis/deterioration at home, embedded into wearable fitness trackers, for example, may prove crucial towards suggesting the possibility of sepsis and more promptly seeking professional help. This is consistent with shifts in healthcare delivery which sees an increasing move towards self-management – where patients and family members are taking a more active role in detecting and managing symptoms, as well in managing medication and taking measurements (39–41). Remote monitoring models accelerated by the coronavirus pandemic (42, 43), and thanks also to technological advances, tech-supported self-testing and monitoring within remote healthcare systems are expanding beyond older adult care and chronic diseases – where the majority of trials focused until recently (44–46). In relation to sepsis, it is important to underline that the symptoms and even the digital vital signs, like heart rate, are non-specific. This reflects onto two potential approaches in relation to home alerts: 1. Generic alerts to seek medical advice, e.g., if heart rate is persistently elevated over time without a clear cause; 2. Development of apps to incorporate both physiological parameters, such as heart rate, and questions about symptoms that might prompt health-seeking behavior – this is not straightforward, however, as the complex hierarchy of questions used by the NHS 111 service demonstrates.

Accordingly, in relation to pre-hospital professional care, our study echoes work which highlighted the usefulness of other digital reminders in GP practices, with the caveat that these must not lead to unnecessary cognitive and care load (47). Reflecting on the crucial role of paramedics, two studies concluded that staff training and advanced point of care tests were more important than giving antibiotics in the ambulance based on patients’ appearance – which did not lead to increased survival (48, 49). However, paired with staff training, testing, and screening tools that could be digitalized, other work found instead that prehospital screening (50) and antibiotics (51) can improve patients’ outcomes.

Participants have echoed the views of professionals in that digital alerts should act as an aid to HCPs, as particularly for those HCPs who are less senior, in busy clinical environments recognizing sepsis can be insidious (19, 52) – as participants in our study commented and recommended. In the hospital, evidence on digital alerts for sepsis/deterioration is growing, yielding the promise of increasing timely treatment and reducing mortality of patients (30, 31). Digital alerts’ positive roles include reminding HCPs to review a patient, supporting decision-making, multidisciplinary teamwork, and facilitating patient’s escalation and team communication (33, 34).

Significantly, this study adds to the current research on sepsis/deterioration digital alerts in the hospital setting the role that they could play in enhancing a clearer communication between patients and HCPs. For example, one of the few studies reporting on survivors’ hospital experience showed that sepsis survivors had positive opinions and feelings of gratefulness for the care they received during hospitalization, as participants in this study also expressed. However, they also felt that improvements were needed. Interestingly, they valued establishing positive interactions with the HCPs on whom they felt deeply dependent (10). Other work has also pointed to challenges in the communication between HCPs and patients – where the former tended to avoid using the term sepsis – and this was found contributing to delays in patients’ care (14, 52). Similarly, in relation to survivors and family members, our study has supported the value of establishing a relationship of mutual trust between patients, their families, and HCPs – where the former feel listened to and informed about the sepsis diagnosis by the latter. The design and use of digital alerts should be guided keeping in mind the crucial importance of a trustworthy doctor-patient relationship – which has been shown to impact patients’ outcomes (53). In this sense, digital alerts might prompt HCPs to share with patients and family members that they are investigating or ruling out sepsis. On the other hand, digital alerts which can factor in patients’ illness experience and views can support the communication with and the decision-making of the clinical team in collaboration with the family members too.

Finally, our study fits with the increasing literature on post-sepsis syndrome (6, 21), and the growing move towards remote monitoring in healthcare (42, 54). Our participants highlighted the need for specialized sepsis aftercare, which others have shown to ameliorate patients’ long-term outcomes, with a significantly improved 5-year survival after suffering from sepsis or septic shock (55). Another work found that primary care post-sepsis intervention reduced post-traumatic stress disorder symptoms after 2 years of stay in the intensive care unit due to sepsis (56). Despite this, sepsis aftercare has been often found inadequate, as in our study (23). Therefore, sepsis clinics, relying on home self-monitoring and digital alerts embedded in remote home monitoring services, are an encouraging promise.

This study reinforced the benefits of exploring the experiences of patients and family members of the sepsis journey to identify potential improvements needed, including untapped potential roles of digital alerts, in promptly identifying, managing, and communicating about sepsis and after-sepsis care (9, 15, 57). The NHS in England, as well as health systems across the world, should aim to conduct recurrent consultations with patient and public contributors. The involvement of patients and family members as key stakeholders in digital health is a growing field, particularly in process evaluations, optimization, and usability testing (57). However, users and patients should be more intensively consulted in setting digital health priorities, both in relation to research agendas and investments in the digital health industry (58, 59). Patients and family members are a key source to learn from about what improvements might be needed that would lead to improved patient outcomes (both physical and psychological) when dealing with sepsis.

Literature relative to the clinical setting has shown how false positive alerts lead to fatigue, desensitization, or over-treatment (60). It is worth highlighting that no existing digital alert for sepsis/deterioration, either rule- or machine learning-based, can be used as a stand-alone diagnostic tool. Additionally, it is important to acknowledge that current digital alerts can only use data that is captured electronically. Many of less common and/or subjective symptoms are not, or not in a way that current alert models can use. There is potential in the medium term to use large language models to read narrative text / notes in the electronic patient record, but this has not yet arrived. As our participants expressed, despite commonalities, digital systems and alerts should indeed become sophisticated and able to factor in patient’s clinical history and experience, in dialogue with a HCP whose judgment remains irreplaceable. Developers and clinical experts, in tight collaboration with survivors and frontline HCPs, should accelerate research in relation to digital alerts at home and how these could be best linked to ambulance, primary and secondary care.

The more the NHS is digitalizing, the more it is connected. However, there are still significant interruptions and a-synchronicity in the flow of data across regions, hospital Trusts, and their digital systems, and between the digital systems of ambulance, primary, and secondary care. Uniformity and standardization are not necessarily the best route to follow, however, this study has indicated the need for a better integrated healthcare system where the journey of a patient becomes less fragmented, both in relation to their clinical history as well as in relation to management and care of single sepsis episodes. Our work adds to the growing evidence in digital healthcare about the necessity for digital transformations to be interoperable and devoted to underpin increasingly integrated healthcare systems (61, 62). Efforts should be made to develop digital technologies, including AI advanced ones, devoted to support the connection of different services, respecting the rights of the patient and the ethics in healthcare (63). Digital transformations, as also our participants commented, are however to be accompanied by organizational improvements, with more resources, staff, education, and effective leadership (61).

Finally, what appears a more immediately viable recommendation of this study is the strengthening of awareness, communication, and support for sepsis sufferers and survivors, particularly in relation to post-sepsis syndrome. In relation to the latter, more routine use of patient information leaflets on discharge is recommended to help inform patients about this and direct them to sources of help and support (64). Digital alerts and easy-to-access resources should better support awareness and the HCP-patient communication, also in sepsis aftercare. Dedicated sepsis teams in all main hospitals across the country, who could perform 24/7 virtual care, both pre- and post-episode, are a possibility that decision-makers should ponder – together with ensuring good level of staffing and resources in the system.

### Strengths and limitations

This study is unique in having explored the experiences of sepsis survivors and family members as well as their views on digital alerts for sepsis/deterioration for HCPs. We recruited a varied sample of participants in terms of age, cause of sepsis, year of sepsis episode, geographical location in England, and type of hospital. The core team includes different disciplinary backgrounds [anthropology (RL), sociology (AlB) and psychology (ST-C)], providing different perspectives and interpretations through regular team discussions, which added rigor to the process of data collection and analysis.

One of the main limitations of the study is the predominance of White British female participants, which hampered the collection of views and experiences from a more demographically varied sample, including ethnic minorities, limiting transferability. Finally, while being less resource-consuming than hospital-based recruitment, social media sampling can be more biased (65).

## Conclusion

Sepsis is a life-threatening disease which can present insidiously and affect survivors with a serious post-episode syndrome. Digital alerts for sepsis/deterioration, if more widely deployed among the general population and across the healthcare system, could be useful along all the phases of the sepsis journey – from onset and help seeking, to hospital and post-sepsis experience. Digital alerts should support the digital transformation of the healthcare system toward greater interoperability and, at the same time, they should be designed with patients and the public, so to factor in their experience-based knowledge and to enhance the doctor-patient communication. Along with increased awareness raising and ongoing education, as well as a sufficiently resourced and staffed health system, digital alerts for sepsis/deterioration have further untapped potential to support better integrated healthcare services, which are increasingly digital, but no less user-centered.

## Data Availability

The raw data supporting the conclusions of this article will be made available by the authors, without undue reservation.

## Footnotes

^1^https://www.imperial.ac.uk/school-public-health/primary-care-and-public-health/research/global-digital-health/research/dials/.
