# Supplementary material for: The sepsis journey and where digital alerts can help: a qualitative, interview study with survivors and family members in England
Source: Front Public Health. 2025 Mar 26;13:1521761. doi: 10.3389/fpubh.2025.1521761 (PMC11995637; doi:10.3389/fpubh.2025.1521761)
Supplement: Supplementary file 1 [file Data_Sheet_1.docx]

# DiAlS Qual Study

**Survivor and Family Member Topic Guide**

## Briefing

1. Welcome and thanks to participant(s) for agreeing to take part.
2. Introduce researcher.
3. This focus group/interview is for the DiAlS study, which aims to explore the views of patients and family members of the management of sepsis in hospitals. Patients will have different experiences of sepsis and different views about how care for patients with sepsis could be improved. Patients’ experiences and views can also vary depending on the sepsis episode and nursing management. We are interested in all experiences and all views.
4. If at any time during the focus group/interview you do not wish to answer a particular question, that’s okay.
5. If you feel that you have difficulty with recollection of your experience with regards to sepsis, please do not worry, we are interested to listen to whatever you remember/feel you can tell us without asking you to give us a whole, detailed picture.
6. We would like to audio record the conversation. The recording will be transcribed, but everything you say will be anonymous. Your name, any names and any places you mention will be taken out, so that if someone read the transcript, they would not know who you are, where you live or which hospital you attended.
7. The focus group discussion/interview will remain confidential.
8. If, at any stage, you wish to stop the audio recording, please let us know. Begin recording.
9. Do you have any questions?

## Topics to be explored

Below is a list of topics to be discussed. The topic guide will remain flexible with respect to what is of importance to participants. All topics and questions apply to either the experience and point of view of the patient or of a family member.

1. Participants’ experience of being ill with sepsis, including different episodes and presentations
2. Participants’ experience of seeking help for (symptoms of) sepsis.
3. Participants’ experience of discussing sepsis (symptoms) with healthcare professionals, particularly in hospital, from initial symptoms to diagnosis and treatment.
4. Participants’ views on how sepsis is identified and managed in hospitals, including on different managements experienced.
5. Participants’ views and experiences of healthcare professionals using (digital) alerts and/or other systems to identify patients with sepsis in hospital.

*Introduction for focus groups: We’d like to start by introducing ourselves and understanding everyone’s previous experience with sepsis, where you are happy to share these experiences. Some people may have had sepsis themselves and some people may have a family member who has had sepsis. Some people may have had sepsis more than once. People may have experienced sepsis very differently and there may be differences in how people were diagnosed and treated. We are interested in all these experiences.

*Introduction for interviews: We would like to start with asking you about your experience in general and then go on to ask some specific questions about the symptoms that you had, how you sought medical help and how you were diagnosed and treated.

If you have had more than one episode of sepsis and each had a different presentation and nursing management, we would be interested in hearing about these different experiences. However, if you have difficulty with recollection, please do not worry.

1. EXPERIENCE IN GENERAL - Could you tell us about when you or your family member was diagnosed with sepsis and briefly what happened?

*Invite each person in the group to give background on their experience with sepsis.*

We’d like to ask some more specific questions about when you/your family member was ill with sepsis.

1. SYMPTOMS - What happened when you were first unwell – as well as on your subsequent episode/s – if you were unwell more than once?
   1. What symptoms did you have?
   2. How long did the symptoms last?
   3. How bad were the symptoms? How much did they interfere with your usual activities?
   4. What did you initially think these symptoms were caused by?
   5. How did you try and manage these symptoms?
2. DECISION TO SEEK HELP - What made you decide to seek medical help, both on the first episode as well as the subsequent ones – if that applies?
   1. What were you most concerned about?
   2. How did you seek medical help? Who did you contact first?
   3. Who else did you seek advice from/ask for support (e.g., family member)?
3. AT ED/HOSPITAL - What happened when you arrived at hospital/A&E, both on the first episode as well as the subsequent ones – if that applies?
   1. What did the healthcare team ask you? What did you tell them about your symptoms?
   2. What observations and tests did they run?
   3. What did the healthcare team explain to you?
   4. How were you monitored whilst you were in A&E?
4. DIAGNOSIS - How were you diagnosed with sepsis, both on the first episode as well as the subsequent ones – if that applies?
   1. Who told you it was sepsis? How was this explained to you?
   2. What alerted doctors that it might be sepsis?
   3. When was sepsis first mentioned? How long did it take to confirm the diagnosis?
   4. How did doctors confirm it was sepsis?
5. TREATMENT - What happened after you were diagnosed with sepsis, both on the first episode as well as the subsequent ones – if that applies?
   1. What initial treatment did you receive?
   2. What later care did you receive?
   3. What happened when you felt better?
6. POSITIVES - What went well in how you were diagnosed and treated, both on the first episode as well as the subsequent ones – if that applies?
   1. **Prompts**: seeking help, time to be seen by doctors/to do investigations, communication with health professionals, consideration of signs and symptoms and previous history, consideration of comorbidities, different roles of healthcare professionals, monitoring whilst in A&E, diagnosis made and communicated, treatment received, care received post-A&E.
7. NEGATIVES - What could have gone better in how you were diagnosed and treated, both on the first episode as well as the subsequent ones – if that applies?
   1. **Prompts**: seeking help, time to be seen by doctors/to do investigations, communication with health professionals, consideration of signs and symptoms and previous history, consideration of comorbidities, different roles of healthcare professionals, monitoring whilst in A&E, diagnosis made and communicated, treatment received, care received post-A&E.

The DiAlS study is carrying out research to help improve the diagnosis and treatment of sepsis in hospitals. The study is particularly interested in the **use of digital alerts**. These are on hospital computers and send a notification to a doctor or nurse that a patient has signs of sepsis.

We are interested in hearing about how you think patients with sepsis could be identified and diagnosed faster.

1. VIEWS ON IDENTIFICATION - What is your understanding of how healthcare teams identify patients with sepsis?
   1. What symptoms do they look for?
   2. What tests do they run?
   3. Which healthcare professionals can identify patients with sepsis?
2. VIEWS ON DIGITAL ALERTS - What do you think about healthcare teams using digital alerts to help identify patients with sepsis?
   1. What information about a patient should be used/inputted in a digital alert? How should they look like?
   2. Which healthcare professionals should use a digital alert for sepsis?
   3. How might a digital alert influence a patient’s care?
   4. What would patients/family members want to know about digital alerts?

[ Before we finish today, we’d just like your final thoughts]

1. Is there anything else that you’d like to mention that you think is important that we haven’t discussed today?
2. Is there something else that we should be asking in future interviews/focus groups?
3. We will be carrying out interviews with hospital doctors to ask about how they care for patients with sepsis. What do you think we should be asking them?

**Demographic questions**

Age range, gender, ethnic background, religious orientation, qualifications, job title
